# Cortical gray-white matter contrast abnormalities in male children with attention deficit hyperactivity disorder

**DOI:** 10.3389/fnhum.2023.1303230

**Published:** 2023-12-21

**Authors:** Changhao Wang, Yanyong Shen, Meiying Cheng, Zitao Zhu, Yuan Lv, Xiaoxue Zhang, Zhanqi Feng, Zhexuan Yang, Xin Zhao

**Affiliations:** ^1^Department of Radiology, The Third Affiliated Hospital of Zhengzhou University, Zhengzhou, China; ^2^Henan International Joint Laboratory of Neuroimaging, Zhengzhou, China; ^3^Medicine Division, Wuhan University, Wuhan, China; ^4^Medical Research Center, The Third Affiliated Hospital of Zhengzhou University, Zhengzhou, Henan, China; ^5^Henan Joint International Laboratory of Glioma Metabolism and Microenvironment Research, Henan Provincial Department of Science and Technology, Zhengzhou, Henan, China; ^6^Institute of Neuroscience, Zhengzhou University, Zhengzhou, Henan, China

**Keywords:** attention deficit hyperactivity disorder, male children, gray-white matter tissue contrast, cortical thickness, inattention

## Abstract

**Purpose:**

Presently, research concerning alterations in brain structure among individuals with attention deficit hyperactivity disorder (ADHD) predominantly focuses on entire brain volume and cortical thickness. In this study, we extend our examination to the cortical microstructure of male children with ADHD. To achieve this, we employ the gray-white matter tissue contrast (GWC) metric, allowing for an assessment of modifications in gray matter density and white matter microstructure. Furthermore, we explore the potential connection between GWC and the severity of disorder in male children by ADHD.

**Methods:**

We acquired 3DT1 sequences from the public ADHD-200 database. In this study, we conducted a comparative analysis between 43 male children diagnosed with ADHD and 50 age-matched male controls exhibiting typical development trajectories. Our investigation entailed assessing differences in GWC and cortical thickness. Additionally, we explored the potential correlation between GWC and the severity of ADHD. To delineate the cerebral landscape, each hemisphere was subdivided into 34 cortical regions using freesurfer 7.2.0. For quantification, GWC was computed by evaluating the intensity contrast of non-normalized T1 images above and below the gray-white matter interface.

**Results:**

Our findings unveiled elevated GWC within the bilateral lingual, bilateral insular, left transverse temporal, right parahippocampal and right pericalcarine regions in male children with ADHD when contrasted with their healthy counterparts. Moreover, the cortical thickness in the ADHD group no notable distinctions that of control group in all areas. Intriguingly, the GWC of left transverse temporal demonstrated a negative correlation with the extent of inattention experienced by male children with ADHD.

**Conclusion:**

Utilizing GWC as a metric facilitates a more comprehensive assessment of microstructural brain changes in children with ADHD. The fluctuations in GWC observed in specific brain regions might serve as a neural biomarker, illuminating structural modifications in male children grappling with ADHD. This perspective enriches our comprehension of white matter microstructure and cortical density in these children. Notably, the inverse correlation between the GWC of the left transverse temporal and inattention severity underscores the potential role of structural and functional anomalies within this region in ADHD progression. Enhancing our insight into ADHD-related brain changes holds significant promise in deciphering potential neuropathological mechanisms.

## 1 Introduction

Attention deficit hyperactivity disorder (ADHD) is one of the most common neurodevelopmental disorders in childhood. The global prevalence of ADHD ranges from 2 to 7%, with an average of about 5% (Sayal et al., 2018), and the incidence in China is 6.5% (Liu et al., 2018). ADHD is often characterized by persistent and age-inappropriate inattention, hyperactivity, impulsivity, varying degrees of learning difficulties, emotional instability, and abnormal behavior, and usually lasts into adulthood (Mak et al., 2020). Therefore, ADHD has a serious impact on children’s life and study. According to the 4th edition of the Diagnostic and Statistical Manual of Mental Disorders (DSM-IV), ADHD can be divided into three subtypes: ADHD-hyperactive type;(ADHD-H), ADHD-inattentive type (ADHD-I) and ADHD-combined type (ADHD-C) (Chhabildas et al., 2001). Recent studies have found that ADHD is more common in males than females (Mowlem et al., 2019). In addition, altered GWC has been found in focal cortical dysplasia (FCD), epilepsy, autism spectrum disorder (ASD), and Alzheimer’s disease (Salat et al., 2011; Blackmon et al., 2015, 2019; Andrews et al., 2017). Studies have shown that in ASD, GWC seems to be caused by the presence of supernumerary neurons beneath the cortical plate, which—in turn—may result from migration deficits or failed apoptosis in the subplate region (Avino and Hutsler, 2010). This finding also agrees with genetic investigations linking the aetiology of ASD to atypical neuronal proliferation, migration, and maturation (Huguet et al., 2013; Pinto et al., 2014). Some studies have indicated that in FCD, the increase in cortical thickness and white matter neuron density may lead to a change in GWC (Sisodiya et al., 2009; Blumcke et al., 2011). ASD and ADHD shared some phenotypic features and have high comorbidity with ADHD (Bethlehem et al., 2017). Existing study has found that children with ADHD have slow development of cortical areas (Shaw et al., 2007), therefore, this study hypothesizes that the GWC of children with ADHD also has abnormal changes and there is a certain relationship between GWC and the severity of ADHD. GWC can reflect the microstructural integrity of white matter and gray matter density of the brain, and evaluate the structural integrity and histological characteristics of the brain, which can be used to study brain diseases and neurodevelopmental abnormalities. To further understand the factors related to GWC, this study analyzed the cortical thickness of ADHD children and the control group and explored whether the cortical thickness changes in brain regions with different GWC.

So far, domestic and foreign scholars have found changes in brain structural integrity in children with ADHD, such as changes in cortical morphology, volume reduction (Hoogman et al., 2017; Albajara Saenz et al., 2019; Yasumura et al., 2019), and reduction or increase in cortical thickness (Almeida Montes et al., 2013; Hoogman et al., 2019). Another study has also found changes in white matter microstructure in ADHD patients using diffusion tensor imaging (DTI) (Hung et al., 2023). In recent years, an increasing number of structural magnetic resonance imaging studies have focused on the boundary between gray and white matter and the GWC in the cortex. Grey-white matter tissue contrast was calculated as a GWC by acquiring gray matter signal intensity at 35% cortical depth and white matter signal intensity at 1.0 mm below the gray-white matter boundary. The higher GWC indicates greater contrast at the gray-white matter boundary. These results may reflect the changes in the microstructure of white matter in ADHD patients, such as increased axons and myelin sheath, which may lead to increased white matter signal intensity and consequently increased GWC. In addition, ADHD patients also have abnormal gray matter density, such as decreased cortical myelin sheath, which may lead to decreased gray matter signal intensity, thereby leading to increased GWC.

It is not clear whether the gender difference in ADHD will affect the brain structure and function of children, and study has found that the incidence of ADHD in males is higher than that in females (Mowlem et al., 2019). Therefore, the present study compared the differences in cortical GWC between male children with ADHD and the matched male healthy control group to explore whether there are specific brain regions with GWC alterations in male children with ADHD. This study also investigated the changes in cortical thickness in male children with ADHD and the association between abnormal GWC changes and the severity of ADHD in male children were analyzed to assess whether these changes could indicate the severity of ADHD. These may be the characteristic changes of ADHD histopathology and may provide more imaging markers for the diagnosis of ADHD, so as to better understand the occurrence of ADHD and provide more help for future diagnosis.

## 2 Materials and methods

### 2.1 Participants

The data used in this study were obtained from the public database ADHD-200.^1^ The ADHD-200 dataset contains functional and anatomical MRI data provided by eight institutions. Each cohort was approved by the research ethics review boards of each institution. All participants or their legal guardians signed informed consent before participating. ADHD-200 database includes data from multiple central sites, but the data only from Peking University was used in our study. The reasons for this decision are as follows: Firstly, considering the variations in scanning sequence parameters of MRI instruments at each center site, which can introduce certain differences in the final results, so this study chose not to conduct a multi-center analysis. Secondly, Peking University had the largest sample size among all center sites and provided relatively detailed questionnaire data.

Due to the higher prevalence of ADHD in male children, this study primarily focused on male children. A total of 245 children’s information was gathered from the Peking University database, comprising 143 in the healthy control group and 102 with ADHD. Inclusion criteria required that participants meet the following conditions: (1) absence of any comorbidities, (2) male children, (3) right-handedness, (4) no other structural brain abnormalities, and (5) had a total score of intelligence scale greater than 80. This initially resulted in the inclusion of 45 male children with ADHD and 80 healthy control male children. However, 2 male children with ADHD were subsequently excluded due to a lack of questionnaire data, resulting in a final inclusion of 43 male children with ADHD. To ensure there were no significant differences in basic characteristics (such as age) between the experimental and control groups, this study selected 50 healthy male children from the 80 healthy control group. Finally, all the children included in this study had an age range of 8−15 years. Psychostimulant medications were withheld at least 48 h prior to scanning. No obvious lesions were found on the cranial MRI plain scan were reports available within the database. ADHD was diagnosed according to the ADHD Rating Scale IV (ADHD-RS-IV), and the intelligence quotient (IQ) was measured by the Chinese Wechsler Intelligence Scale for Children -Revised. The severity of ADHD symptoms was assessed using the ADHD Rating Scale-IV (ADHD-RS-IV), with higher scores indicating greater severity of ADHD. The demographic data and clinical variables of this study are presented in Table 1.

**TABLE 1:** Participant characteristics.

	ADHD *n* = 43 ($\bar{\text{x}}\pm \text{s}$)/ M(Q_R_)	HC *n* = 50 ($\bar{\text{x}}\pm \text{s}$)/ M(Q_R_)	*T* value/*Z* value	*P* value
Age IQ	11.08 (9.83, 13.17)	12.25 (10.02, 13.50)	1.210	0.226
Verbal	114.02 ± 17.32	119.33 ± 14.38	−1.605	0.112
Performance	101.00 (92.00, 106.00)	112.61 ± 12.63	4.547	<0.000*
Full-scale ADHD-RS	108.58 ± 13.12	117.13 ± 13.27	−3.482	0.001*
Inattention score	27.53 ± 3.69	14.75 (13.00, 17.00)	−7.827	<0.000*
Hyperactivity/impulsivity score	23.00 (17.00, 25.00)	12.50 (11.00, 16.00)	−6.537	<0.000*
Total score	49.56 ± 7.78	28.94 ± 5.80	14.202	<0.000*

*Significant at *p* < 0.05.

ADHD, attention-deficit/hyperactivity disorder; HC, healthy control; ADHD-RS, ADHD Rating Scale.

### 2.2 Image processing

While the database mentioned that all participating children had no history of other neurological disorders or head injuries, information from the Peking University database revealed that some children still had structural abnormalities, such as a cyst in the left temporal pole. In the interest of thoroughness and caution, this study engaged two experienced radiologists to assess 93 cranial MRI plain scan images of the children from the Peking University database, in order to mitigate the influence of subjective factors when assessing image quality and to obtain reliable experimental data, it is required that doctors remain blinded to the conditions of children with ADHD and healthy control children during the evaluation process. This assessment was performed to identify any significant structural abnormalities, ensuring the quality and accuracy of subsequent image segmentation. The 3DT1 sequences in the ADHD-200 database were used for image processing. The freesurfer 7.2.0 software was used for pre-processing, including image scalping, Talairach transformation, gray matter surface reconstruction, and subcortical structure segmentation. In order to ensure the quality of the images, motion correction was performed on all images. Gray matter and white matter signal intensities were sampled on non-uniform normalized images (nu.mgz). The gray matter signal intensity was collected at 35% of the cortical depth distance from the white matter boundary, and the white matter signal intensity was collected at 1 mm below the white matter boundary, then the formula was used to calculate the GWC.

$$GWC(\frac{100\,\left(W\,M\,I_{1\,M\,M}-G\,M\,I_{35\%}\right)}{0.5\,\left(W\,M\,I_{1\,M\,M}+G\,M\,I_{35\%}\right)}$$

Finally, the mri_segstats function was used to extract the GWC of 34 brain regions in the both cerebral hemispheres according to the Desikan-Kiliany atlas. Cortical thickness was measured as the shortest distance from the white matter at each vertex to the cortical surface.

### 2.3 Statistical analysis

The data were analyzed using the SPSS.26 software. The information (such as age, intelligence quotient, ADHD-RS-IV, etc.) of children in ADHD group and healthy control group were analyzed. GWC and cortical thickness in 68 cortical regions of interest were compared between the ADHD group and the healthy control group. Data conformed to the normal distribution were analyzed using student’s *t*-tests, and the data were presented as mean ± standard deviation (SD). Data that did not follow the normal distribution were analyzed by Mann-Whitney *U* test and represented as M(Q_*R*_). The brain regions with differing GWC values in the children with ADHD were subjected to a correlation analysis with the ADHD Rating Scale-IV (ADHD-RS-IV). For metric data following a normal distribution, Pearson correlation analysis was employed, while for non-normally distributed metric data, Spearman correlation analysis was used. A *p* value <0.05 was statistically significant. Bonferroni adjustment was applied to GWC and cortical thickness comparisons across 34 cortical regions of interest (*p* < 0.00147).

## 3 Results

### 3.1 Participants

There was no significant difference in age between male children with ADHD and the corresponding healthy male control group participants in the study. However, substantial distinctions emerged in terms of IQ levels and the severity of ADHD symptoms, as highlighted in Table 1. The evaluation of IQ revealed no statistically significant divergence in Verbal scores between the ADHD group and the healthy control group.

### 3.2 Abnormal gray-white matter contrast

**FIGURE 1:**
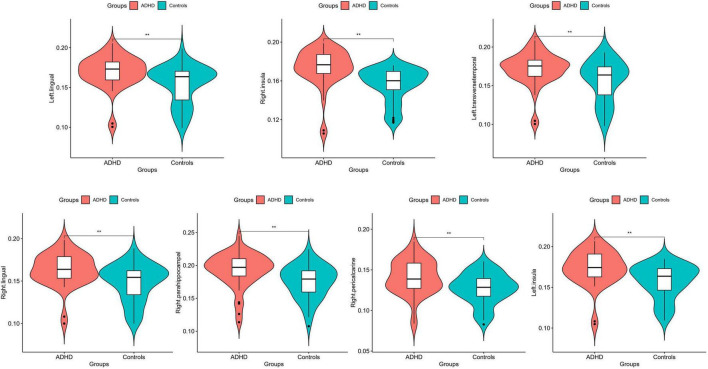
Average quantitative comparisons of GWC differences between ADHD and HC within specific brain regions. **Significant at *p* < 0.00147.

**FIGURE 2:**
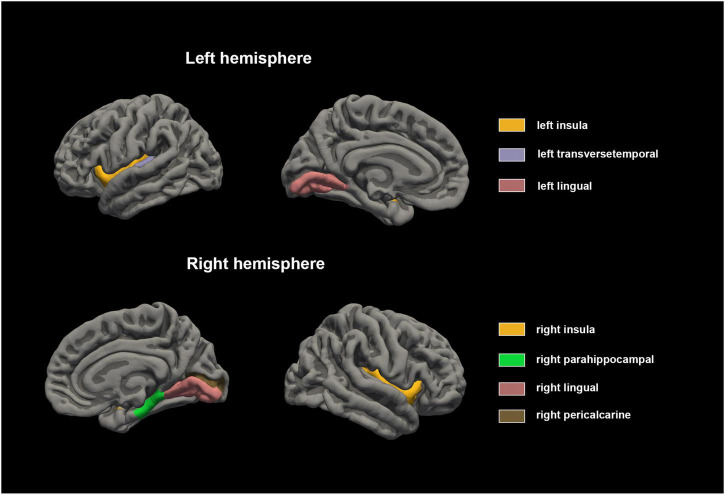
Cortical gray matter contrast abnormalities in male children with ADHD compared with healthy control. The results of Mann-Whitney *U* test showed that the GWC values of bilateral lingual, bilateral insular, left transverse temporal, right parahippocampal and right pericalcarine regions were higher in male children with ADHD.

Statistical analysis was conducted on the GWC of distinct brain regions within male children diagnosed with ADHD and the male control group of healthy individuals, as detailed in Table 2. The results revealed noteworthy disparities, wherein the GWC in the bilateral lingual, bilateral insular, left transverse temporal, right parahippocampal and right pericalcarine regions of the ADHD group exceeded those observed in the male healthy control group (*p* < 0.00147). Conversely, no significant distinctions were identified in the remaining brain regions (as depicted in Figures 1, 2). These results conclusively underscore heightened GWC in the bilateral lingual, bilateral insular, left transverse temporal, right parahippocampal and right pericalcarine areas, with the most pronounced contrast emerging precisely at the interface between gray and white matter.

**TABLE 2:** Group comparisons of GWC.

	Brain region	ADHD *n* = 43 ($\bar{\text{x}}\pm \text{s}$)/ M(Q_R_)	HC *n* = 50 ($\bar{\text{x}}\pm \text{s}$)/ M(Q_R_)	*T* value/*Z* value	*P* value	Power
**GWC from cortical regions ipsilateral**						
1	L-bankssts	0.24 (0.22, 0.25)	0.23 (0.19, 0.25)	−1.637	0.10153	0.46837
2	L-caudalanteriorcingulate	0.24 (0.22, 0.26)	0.23 (0.19, 0.24)	−2.485	0.01295	0.78159
3	L-caudalmiddlefrontal	0.23 (0.22, 0.24)	0.22 (0.19, 0.23)	−2.015	0.04390	0.48571
4	L-cuneus	0.17 (0.16, 0.19)	0.16 (0.13, 0.17)	−2.616	0.00889	0.81617
5	L-entorhinal	0.17 ± 0.03	0.16 ± 0.02	2.804	0.00616	0.78548
6	L-fusiform	0.21 (0.20, 0.22)	0.21 (0.17, 0.22)	−2.462	0.01382	0.78644
7	L-Inferiorparietal	0.22 (0.21, 0.23)	0.22 (0.17, 0.23)	−2.134	0.03280	0.62789
8	L-inferiortemporal	0.23 (0.22, 0.24)	0.22 (0.19, 0.23)	−2.570	0.01017	0.72543
9	L-isthmuscingulate	0.20 (0.19, 0.21)	0.19 (0.16, 0.20)	−2.373	0.01763	0.80279
10	L-lateraloccipital	0.19 (0.18, 0.20)	0.19 (0.15, 0.19)	−2.131	0.03312	0.68966
11	L-lateralorbitofrontal	0.21 (0.20, 0.22)	0.21 (0.18, 0.22)	−2.331	0.01975	0.72635
12	L-lingual	0.17 (0.16, 0.18)	0.16 (0.13, 0.17)	−3.337	0.00085**	0.92245
13	L-medialorbitofrontal	0.2 (0.19, 0.21)	0.20 (0.17, 0.21)	−0.686	0.49283	0.27715
14	L-middletemporal	0.23 (0.21, 0.24)	0.22 (0.19, 0.23)	−2.840	0.00452	0.79204
15	L-parahippocampal	0.19 ± 0.03	0.18 ± 0.03	3.213	0.00181	0.89911
16	L-paracentral	0.19 (0.18, 0.20)	0.18 (0.14, 0.20)	−2.011	0.04430	0.61606
17	L-parsopercularis	0.23 (0.22, 0.24)	0.22 (0.19, 0.23)	−3.059	0.00222	0.76518
18	L-parsorbitalis	0.22 (0.21, 0.24)	0.21 (0.19, 0.22)	−2.620	0.00879	0.76446
19	L-parstriangularis	0.23 (0.22, 0.24)	0.23 (0.18, 0.23)	−2.878	0.00400	0.71754
20	L-pericalcarine	0.15 (0.13, 0.17)	0.14 (0.12, 0.15)	−3.125	0.00178	0.91108
21	L-postcentral	0.19 (0.18, 0.20)	0.18 (0.15, 0.19)	−2.605	0.00920	0.72266
22	L-posteriorcingulate	0.22 (0.21, 0.24)	0.21 (0.18, 0.22)	−2.743	0.00608	0.77006
23	L-precentral	0.20 (0.19, 0.21)	0.19 (0.16, 0.20)	−2.431	0.01505	0.60938
24	L-precuneus	0.21 (0.20, 0.22)	0.21 (0.16, 0.22)	−2.081	0.03747	0.72708
25	L-rostralanteriorcingulate	0.20 (0.20, 0.23)	0.20 (0.17, 0.21)	−2.805	0.00503	0.72690
26	L-rostralmiddlefrontal	0.23 (0.22, 0.25)	0.23 (0.20, 0.24)	−1.618	0.10561	0.49512
27	L-superiorfrontal	0.23 (0.22, 0.24)	0.22 (0.19, 0.24)	−1.980	0.04766	0.54655
28	L-superiorparietal	0.21 (0.20, 0.22)	0.20 (0.16, 0.22)	−1.969	0.04897	0.60658
29	L-superiortemporal	0.22 (0.21, 0.23)	0.21 (0.18, 0.22)	−2.963	0.00305	0.77592
30	L-supramarginal	0.23 (0.22, 0.24)	0.22 (0.18, 0.23)	−2.454	0.01411	0.61439
31	L-frontalpole	0.22 (0.20, 0.23)	0.21 (0.19, 0.23)	−1.241	0.21475	0.29766
32	L-temporalpole	0.19 (0.18, 0.22)	0.19 (0.16, 0.20)	−2.447	0.01442	0.79096
33	L-transversetemporal	0.18 (0.16, 0.18)	0.16 (0.13, 0.17)	−3.190	0.00142**	0.84398
34	L-insula	0.17 (0.16, 0.19)	0.16 (0.14, 0.17)	−3.772	0.00016**	0.96163
**GWC from cortical regions contralateral**						
1	R-bankssts	0.23 (0.22, 0.25)	0.22 (0.19, 0.23)	−2.836	0.00457	0.84873
2	R-caudalanteriorcingulate	0.23 (0.22, 0.25)	0.23 (0.19, 0.24)	−1.422	0.15510	0.48444
3	R-caudalmiddlefrontal	0.22 (0.21, 0.23)	0.22 (0.18, 0.23)	−1.530	0.12612	0.46648
4	R-cuneus	0.16 (0.15, 0.18)	0.16 (0.13, 0.17)	−2.655	0.00794	0.82395
5	R-entorhinal	0.17 ± 0.02	0.16 (0.14, 0.17)	−2.685	0.00724	0.83436
6	R-fusiform	0.20 (0.19, 0.21)	0.19 (0.17, 0.20)	−3.175	0.00150	0.86250
7	R-Inferiorparietal	0.21 (0.20, 0.23)	0.21 (0.18, 0.22)	−2.146	0.03187	0.61274
8	R-inferiortemporal	0.21 (0.20, 0.22)	0.20 (0.18, 0.21)	−2.932	0.00337	0.80320
9	R-isthmuscingulate	0.19 (0.18, 0.20)	0.18 (0.16, 0.19)	−2.824	0.00474	0.84535
10	R-lateraloccipital	0.18 (0.18, 0.20)	0.18 (0.16, 0.19)	−2.319	0.02037	0.71611
11	R-lateralorbitofrontal	0.21 (0.20, 0.22)	0.20 (0.17, 0.21)	−1.930	0.05357	0.93258
12	R-lingual	0.16 (0.15, 0.18)	0.15 (0.13, 0.16)	−3.329	0.00087**	0.33749
13	R-medialorbitofrontal	0.20 (0.19, 0.21)	0.20 (0.18, 0.21)	−0.971	0.33158	0.72330
14	R-middletemporal	0.22 (0.21, 0.23)	0.21 (0.19, 0.22)	−2.643	0.00821	0.90728
15	R-parahippocampal	0.20 (0.18, 0.21)	0.18 ± 0.02	−3.444	0.00057**	0.63640
16	R-paracentral	0.20 (0.18, 0.20)	0.18 (0.15, 0.20)	−2.431	0.01505	0.48532
17	R-parsopercularis	0.22 (0.21, 0.24)	0.22 (0.19, 0.23)	−2.420	0.01554	0.67419
18	R-parsorbitalis	0.22 (0.20, 0.24)	0.21 (0.18, 0.23)	−1.980	0.04766	0.58825
19	R-parstriangularis	0.23 (0.21, 0.24)	0.22 (0.18, 0.23)	−2.670	0.00758	0.72727
20	R-pericalcarine	0.14 ± 0.02	0.13 ± 0.02	3.502	0.00071**	0.91492
21	R-postcentral	0.18 (0.17, 0.20)	0.17 ± 0.03	−2.643	0.00822	0.69103
22	R-posteriorcingulate	0.22 (0.21, 0.23)	0.21 (0.17, 0.22)	−2.867	0.00415	0.84347
23	R-precentral	0.19 (0.18, 0.20)	0.18 (0.15, 0.20)	−1.946	0.05169	0.55017
24	R-precuneus	0.21 (0.20, 0.22)	0.20 (0.16, 0.21)	−1.992	0.04638	0.71842
25	R-rostralanteriorcingulate	0.22 (0.20, 0.23)	0.22 (0.19, 0.23)	−1.765	0.07763	0.59980
26	R-rostralmiddlefrontal	0.23 (0.22, 0.25)	0.23 (0.19, 0.24)	−1.772	0.07634	0.47673
27	R-superiorfrontal	0.23 (0.22, 0.24)	0.23 (0.19, 0.24)	−1.838	0.06609	0.56487
28	R-superiorparietal	0.20 (0.19, 0.21)	0.20 (0.16, 0.21)	−1.726	0.08433	0.56397
29	R-superiortemporal	0.21 (0.20, 0.22)	0.20 (0.19, 0.22)	−2.751	0.00594	0.72244
30	R-supramarginal	0.21 (0.20, 0.23)	0.21 (0.18, 0.22)	−1.699	0.08930	0.50086
31	R-frontalpole	0.21 (0.20, 0.23)	0.21 (0.19, 0.22)	−0.936	0.34914	0.18125
32	R-temporalpole	0.19 ± 0.02	0.18 ± 0.02	2.833	0.00567	0.82794
33	R-transversetemporal	0.16 (0.15, 0.18)	0.15 (0.12, 0.17)	−2.824	0.00474	0.76951
34	R-insula	0.18 (0.17, 0.19)	0.16 (0.15, 0.17)	−4.993	0.00001**	0.99511

**Significant at Bonferroni adjusted threshold of *p* < 0.00147.

ADHD, attention-deficit/hyperactivity disorder; HC, healthy control. L and R denote the right hemisphere and the left hemisphere, respectively.

This study conducted a power analysis for the results of 34 brain regions, and it was found that in the above brain regions in which differential GWC between groups the left lingual exhibited a power of 0.92245, the left transverse temporal had a power of 0.84398, and the left insula showed a power of 0.96163. On the right side, the power for the right lingual was 0.33749, for the right parahippocampal was 0.63640, for the right pericalcarine was 0.91492, and for the right insula was 0.99511. Among them, powers greater than 0.8 for the left lingual, left transverse temporal, left insula, right pericalcarine, and right insula indicate high power, suggesting a robust study design capable of detecting true effects and reducing the risk of Type II errors. The power for the right parahippocampal, falling between 0.5 and 0.8, signifies medium power, indicating a high probability of detecting true effects. A power below 0.5 for the right lingual suggests potential instability in detecting true effects, signifies low power, possibly necessitating a larger sample size in future studies to enhance the reliability of the research.

### 3.3 Abnormal cortical thickness

The computation of cortical thickness for distinct brain regions was performed in both male children diagnosed with ADHD and the male control group comprising healthy individuals. This data, as presented in Table 3, underwent an analysis using the S-W test to ascertain its adherence to a normal distribution. Subsequently, student’s *t* test and Wilcoxon rank-sum test were administered.

**TABLE 3:** Group comparisons of cortical thickness.

	Brain region	ADHD *n* = 43 ($\bar{\text{x}}\pm \text{s}$)/ M(Q_R_)	HC *n* = 50 ($\bar{\text{x}}\pm \text{s}$)/ M(Q_R_)	*T* value/ *Z* value	*P* value	Power
**CT from cortical regions ipsilateral**						
1	L-bankssts	2.64 ± 0.16	2.61 ± 0.19	0.728	0.46865	0.11118
2	L-caudalanteriorcingulate	2.68 ± 0.19	2.63 ± 0.19	1.256	0.21247	0.23718
3	L-caudalmiddlefrontal	2.74 ± 0.11	2.72 ± 0.1	0.633	0.52806	0.09607
4	L-cuneus	1.97 ± 0.12	1.96 ± 0.17	0.264	0.79246	0. 05784
5	L-entorhinal	3.23 ± 0.3	3.28 ± 0.28	−0.838	0.40411	0.13174
6	L-fusiform	2.79 ± 0.1	2.79 ± 0.13	−0.122	0.90310	0.05167
7	L-Inferiorparietal	2.61 ± 0.1	2.62 ± 0.11	−0.263	0.79279	0.05781
8	L-inferiortemporal	2.87 (2.81, 2.93)	2.84 (2.72, 2.96)	−1.210	0.22634	0.31350
9	L-isthmuscingulate	2.37 (2.33, 2.44)	2.38 ± 0.11	−0.243	0.80820	0.05456
10	L-lateraloccipital	2.26 ± 0.1	2.26 ± 0.11	0.167	0.86759	0.05314
11	L-lateralorbitofrontal	2.89 ± 0.15	2.85 ± 0.13	1.349	0.18055	0.28065
12	L-lingual	2.17 ± 0.12	2.09 ± 0.16	2.858	0.00529	0.26654
13	L-medialorbitofrontal	2.65 ± 0.14	2.61 ± 0.13	1.574	0.11895	0.80717
14	L-middletemporal	2.98 ± 0.14	2.98 (2.85, 3.09)	−0.208	0.83518	0.34384
15	L-parahippocampal	2.72 ± 0.24	2.67 ± 0.18	1.216	0.22729	0.11786
16	L-paracentral	2.64 ± 0.15	2.62 ± 0.12	0.594	0.55432	0.22523
17	L-parsopercularis	2.79 ± 0.12	2.79 ± 0.11	0.026	0.97913	0.09034
18	L-parsorbitalis	2.94 ± 0.18	2.9 ± 0.21	0.919	0.36053	0.05007
19	L-parstriangularis	2.73 ± 0.15	2.7 ± 0.12	0.729	0.46797	0.14876
20	L-pericalcarine	1.71 (1.64, 1.79)	1.70 (1.59, 1.87)	−0.435	0.66328	0.11137
21	L-postcentral	2.2 ± 0.12	2.18 ± 0.14	0.569	0.57080	0.07176
22	L-posteriorcingulate	2.6 ± 0.12	2.62 ± 0.14	−0.500	0.61838	0.08701
23	L-precentral	2.66 ± 0.12	2.69 (2.56, 2.76)	−0.447	0.65491	0.07846
24	L-precuneus	2.55 ± 0.11	2.55 ± 0.12	−0.141	0.88797	0.05042
25	L-rostralanteriorcingulate	2.91 ± 0.18	2.8 ± 0.21	2.733	0.00753	0.05224
26	L-rostralmiddlefrontal	2.62 ± 0.13	2.61 ± 0.11	0.413	0.68089	0.77170
27	L-superiorfrontal	2.95 (2.90, 3.06)	2.97 ± 0.12	−0.420	0.67450	0.06929
28	L-superiorparietal	2.38 ± 0.1	2.37 ± 0.1	0.360	0.7198	0.10961
29	L-superiortemporal	2.96 ± 0.14	2.9 ± 0.14	1.815	0.07283	0.06464
30	L-supramarginal	2.66 ± 0.12	2.66 ± 0.1	−0.089	0.92903	0.43487
31	L-frontalpole	3.07 ± 0.28	3.1 ± 0.28	−0.579	0.56373	0.05089
32	L-temporalpole	3.57 (3.41, 3.77)	3.59 ± 0.21	−0.231	0.81718	0.08842
33	L-transversetemporal	2.61 ± 0.17	2.54 ± 0.19	2.026	0.04567	0.09476
34	L-insula	3.13 ± 0.17	3.06 ± 0.15	2.333	0.02187	0.51789
**CT from cortical regions contralateral**						
1	R-bankssts	2.77 ± 0.17	2.71 ± 0.17	1.619	0.10895	0.63610
2	R-caudalanteriorcingulate	2.54 ± 0.21	2.54 ± 0.19	0.045	0.96408	0.36026
3	R-caudalmiddlefrontal	2.71 ± 0.13	2.72 ± 0.11	−0.423	0.67353	0.05022
4	R-cuneus	2.05 (1.96, 2.10)	2.02 (1.91, 2.14)	−1.029	0.30360	0.07026
5	R-entorhinal	3.36 (3.12, 3.52)	3.37 (3.21, 3.58)	−0.844	0.39880	0.30843
6	R-fusiform	2.84 ± 0.11	2.81 ± 0.14	0.990	0.32479	0.14024
7	R-Inferiorparietal	2.64 ± 0.11	2.64 ± 0.1	−0.137	0.89098	0.16509
8	R-inferiortemporal	2.91 ± 0.11	2.87 ± 0.14	1.534	0.12849	0.05212
9	R-isthmuscingulate	2.34 ± 0.14	2.36 (2.28, 2.42)	−0.624	0.53252	0.32944
10	R-lateraloccipital	2.33 ± 0.11	2.33 ± 0.12	−0.057	0.95468	0.08646
11	R-lateralorbitofrontal	2.8 ± 0.14	2.79 ± 0.15	0.393	0.69534	0.05036
12	R-lingual	2.18 (2.12, 2.27)	2.17 (2.04, 2.24)	−1.295	0.19546	0.06748
13	R-medialorbitofrontal	2.64 ± 0.16	2.64 ± 0.13	−0.014	0.98849	0.33869
14	R-middletemporal	3.01 ± 0.14	2.98 ± 0.19	0.797	0.42774	0.05002
15	R-parahippocampal	2.69 ± 0.22	2.63 ± 0.17	1.404	0.16387	0.12364
16	R-paracentral	2.63 ± 0.13	2.59 ± 0.13	1.731	0.08688	0.28431
17	R-parsopercularis	2.82 ± 0.15	2.8 ± 0.12	0.591	0.55621	0.40240
18	R-parsorbitalis	2.88 ± 0.19	2.84 ± 0.19	1.038	0.30208	0.08995
19	R-parstriangularis	2.70 (2.61, 2.77)	2.69 ± 0.11	−0.459	0.64659	0.17680
20	R-pericalcarine	1.74 ± 0.15	1.71 ± 0.18	1.000	0.31982	0.09971
21	R-postcentral	2.19 (2.11, 2.27)	2.2 ± 0.16	−0.170	0.86538	0.16756
22	R-posteriorcingulate	2.53 ± 0.12	2.50 (2.42, 2.57)	−0.116	0.31460	0.05161
23	R-precentral	2.66 (2.52, 2.69)	2.65 (2.55, 2.70)	−0.150	0.88055	0.09668
24	R-precuneus	2.55 ± 0.11	2.56 ± 0.09	−0.376	0.70752	0.05003
25	R-rostralanteriorcingulate	2.85 ± 0.2	2.82 ± 0.17	0.835	0.40569	0.06603
26	R-rostralmiddlefrontal	2.58 ± 0.11	2.59 ± 0.11	−0.464	0.64374	0.13118
27	R-superiorfrontal	2.92 (2.84, 2.99)	2.92 ± 0.12	−0.200	0.84120	0.07447
28	R-superiorparietal	2.38 ± 0.1	2.37 ± 0.09	0.328	0.74403	0.06011
29	R-superiortemporal	2.99 ± 0.15	2.95 ± 0.14	1.171	0.24482	0.21225
30	R-supramarginal	2.69 ± 0.12	2.69 ± 0.14	−0.059	0.9528	0.05039
31	R-frontalpole	3 ± 0.29	3.04 (2.92, 3.18)	−0.913	0.36117	0.18843
32	R-temporalpole	3.64 (3.43, 3.80)	3.63 ± 0.27	−0.123	0.90188	0.06168
33	R-transversetemporal	2.57 ± 0.18	2.56 ± 0.2	0.425	0.67189	0.07048
34	R-insula	3.14 ± 0.18	3.08 ± 0.15	1.963	0.05274	0.49284

ADHD, attention-deficit/hyperactivity disorder; HC, healthy control; CT, cortical thickness. L and R denote the right hemisphere and the left hemisphere, respectively.

The outcomes disclosed that the cortical thickness in the ADHD group no notable distinctions that of control group in all areas (*p* > 0.00147).

### 3.4 Clinical correlates of GWC abnormalities in left transverse temporal

The above-mentioned brain regions in which differential GWC between groups were investigated in relation to the severity of ADHD, we conducted a correlation analysis between GWC corresponding to different brain regions and scores from the ADHD-RS-IV scale, as presented in Table 4. The results of this analysis suggested that the absence of any significant correlation between GWC and the ADHD-RS-IV scale within bilateral lingual, bilateral insular, right parahippocampal and right pericalcarine lobes.

**TABLE 4:** Correlation between GWC and ADHD severity.

		ADHD-Index	Inattentive	Hyper-Impulsive
L-lingual (Spearman)	*r* value	−0.08	−0.128	−0.026
	*p* value	0.608	0.415	0.871
	power	0.080	0.129	0.052
L-transverse temporal (Spearman)	*r* value	−0.161	−0.332	−0.011
	*p* value	0.301	0.03*	0.944
	power	0.179	0.596	0.050
L-insula (Spearman)	*r* value	−0.123	−0.286	0.014
	*p* value	0.432	0.063	0.929
	power	0.123	0.467	0.050
R-lingual (Spearman)	*r* value	−0.107	−0.138	−0.051
	*p* value	0.496	0.377	0.743
	power	0.105	0.143	0.061
R-parahippocampal (Spearman)	*r* value	−0.136	−0.17	−0.079
	*p* value	0.384	0.275	0.614
	power	0.140	0.194	0.079
R-pericalcarine (Pearson)	*r* value	−0.172	−0.277	−0.100
	*p* value	0.269	0.073	0.525
	power	0.198	0.443	0.097
R-insula (Spearman)	*r* value	−0.125	−0.164	−0.069
	*p* value	0.425	0.294	0.662
	power	0.126	0.184	0.072

*Significant at *p* < 0.05.

ADHD, attention-deficit/hyperactivity disorder. L and R denote the right hemisphere and the left hemisphere, respectively.

**FIGURE 3:**
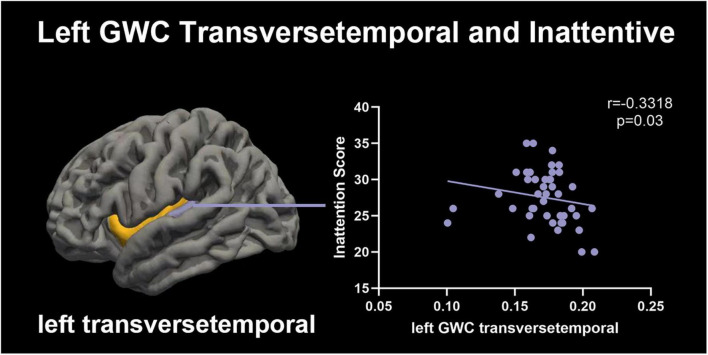
Correlation between left transverse temporal GWC values and inattention. The contrast in cortical grey-white matter tissue exhibited an inverse relationship with the scores on the Inattention scale, particularly observed in the ipsilateral hemisphere of male children diagnosed with ADHD and featuring left transverse temporal involvement.

An intriguing finding emerged regarding the GWC of the left transverse temporal region, which exhibited a negative correlation with the inattentive component of the ADHD-RS-IV (*r* = −0.332, *p* = 0.03), the left insula showed a power of 0.596, as illustrated in Figure 3. These results robustly demonstrated that a more conspicuous gray-white matter contrast within the left transverse temporal region corresponded to a lower degree of inattention among children diagnosed with ADHD.

However, in the correlation analysis, no *p*-value <0.00147 was found, suggesting that the correlation with *p* = 0.03 may not be particularly robust. Nevertheless, the Bonferroni correction is stringent and may lead to an increased risk of false negatives, especially in situations with limited sample size. In our correlation study, the sample size is relatively small, comprising only 43 ADHD children with the scale applied. Applying strict correction methods may potentially obscure genuine effects.

## 4 Discussion

Our study illuminates several key findings. When compared with the male healthy control group, male children diagnosed with ADHD exhibited higher GWC in bilateral lingual, bilateral insular, left transverse temporal, right parahippocampal and right pericalcarine regions. Moreover, a noteworthy inverse correlation was detected between the GWC of the left transverse temporal and the inattentive score as assessed by ADHD-RS-IV (Table 4). The GWC calculation formula suggests that elevated GWC coincide with an augmented ratio of WMI to GMI. This shift may denote intensified white matter signal intensity or diminished gray matter signal intensity. These changes might signify variations in cortical myelin, cortical thickness, gray matter volume, or even microstructural alterations such as white matter axons and myelin. Further, our analysis of cortical thickness in ADHD group no notable distinctions that of control group in all areas.

Importantly, all the aforementioned cortical regions are intricately linked to the distinctive symptoms of ADHD. For instance, the parahippocampal region, a component of the temporal lobe, functions as a pivotal memory and spatial navigation center. Concurrently, it forms an intricate memory network encompassing regions like the hippocampus, parahippocampal, amygdala, and prefrontal cortex, culminating in higher cognitive processes including memory and emotional processing (Squire et al., 2004). Similarly, the insula, a limbic system component, exerts motor control and is implicated in self-regulation and emotional control, areas often impaired in ADHD (Norman et al., 2016). Lingual area is primarily located at the bottom of the occipital lobe. It is involved in the processing of visual information in the human body and may be associated with reading, particularly in the processing of written language and words (Fink et al., 1996). Pericalcarine region is located between the parietal lobe and the temporal lobe. It is generally believed to be involved in reading, understanding written language, and particularly in the process of integrating visual and language information. The transverse temporal area, houses within the temporal lobe, serves as a vital auditory and language center, engaged in the processing and comprehending auditory and linguistic stimuli. It collaborates with various other brain regions, such as the temporo-parietal association cortex, amygdala, hippocampus, parahippocampal region, and prefrontal cortex, contributing to sophisticated cognitive functions encompassing hearing, memory, emotion, and language. Prior studies have noted abnormal function in the right temporal lobe in ADHD, reinforcing its role in memory and executive functions (Gu et al., 2018).

Alterations in white matter signal intensity potentially stem from changes in white matter microstructure. White matter predominantly comprises neuronal axons and myelin, both rich in lipids. Consequently, the signal intensity of white matter hinges on lipid content and arrangement. An elevated number of axons and myelin within white matter neurons increases lipid content, heightening white matter signal intensity. Studies using diffusion tensor imaging (DTI) have shown that variations in white matter nerve fibers orientation directly influence water molecule diffusion and fractional anisotropy (FA) in DTI, reflecting changes in nerve fiber density, diameter, and myelin composition (Beaulieu, 2002). Notably, one study has found that male children with ADHD had increased FA values in the left fronto-temporal regions and parieto-occipital regions compared with healthy controls (Silk et al., 2009). Besides, another study has found that FA value in the white matter of temporal lobe was elevated in adults with ADHD (Konrad et al., 2010). Peterson et al. (2011) also found a significant increase in the FA value of the parahippocampal through DTI.

In an age-related GWC study, it was found that decreased GWC may reflect increased water content in white matter as well as increased neuronal loss in gray matter (Magnaldi et al., 1993). In addition, some scholars believed that the reduction of GWC was related to the change of the degree of myelination (Vidal-Pineiro et al., 2016). Myelination of neuronal fibers is essential in the development of the brain and is a key factor in the transmission of neural signals in the brain. Myelin formation promotes nerve growth and axonal extension through oligodendrocyte dependent myelin production. It has been suggested that the dysregulation of myelin may be the development mechanism of ADHD (Lesch, 2019). Despite the high content of myelin in white matter, some studies have found that there is still a high content of myelin in the cortex (Edwards et al., 2018). The signal intensity of gray and white matter is closely related to the content of myelin (Yasuno et al., 2017). Although no studies have demonstrated that myelin may be a direct factor of GWC, changes in GWC are sensitive to ADHD-related myelination. T2 relaxation values are mainly affected by tissue content of myelin (Laule et al., 2007). The damage of gray matter area or the change of microstructure may be the pathophysiological basis of the abnormal structure or function. In integrated magnetic resonance imaging, some researchers found that the T2 relaxation value of temporal gray matter in children with ADHD was higher, and the increase of T2 relaxation value reflected the decreased content of cerebral myelin and the corresponding increased content of water (Deoni et al., 2015). The decreased content of myelin in the brain may be caused by factors such as destruction, loss or reduction of myelin, and these changes may lead to a decrease in the conduction velocity of nerve impulses and thus affect the signal intensity of gray matter (Bartzokis et al., 2010; Nave and Werner, 2014). At present, the microstructure that influences GWC has not been fully determined, but some factors may play a role in it. For example, changes in water content caused by gray matter shrinkage lead to changes in gray matter signal intensity, and demyelination also causes changes in water content resulting in changes of gray/white matter signal intensity, both of the above factors can lead to changes of GWC (Magnaldi et al., 1993).

Cortical thickness and brain volume stand as the primary metrics for discerning the intricacies of brain microstructure. The reduction in cortical thickness and gray matter volume can be attributed to factors such as neurons and synapses loss, as well as neuronal atrophy or degeneration. These alterations can subsequently result in changes in gray matter density, potentially contributing to anomalous fluctuations in GWC. In a study about Down syndrome, the researchers found that brain regions with altered cortical thickness partly overlapped with regions with altered GWC in people with Down syndrome, which indicated that some of the differences in cortical thickness in Down syndrome may be driven by the differences in GWC (Bletsch et al., 2018). At present, many studies have found that the overall brain volume of ADHD patients decreases by 3−5%, and gray matter is affected first (Greven et al., 2015), and the cortical thickness of frontal lobe, temporal lobe and insular lobe were also decreased (Hoogman et al., 2019; Maier et al., 2023). In terms of gray matter volume, studies have found that ADHD patients have reduced cortical gray matter volume in the right basal ganglia and insula (Norman et al., 2016), and the gray matter volume in the amygdala and hippocampus were also decreased (Yu et al., 2022). Furthermore, in a study on gender differences, it was found that the gray matter volume of the left lingual gyrus is reduced in male children with ADHD (Gonchigsuren et al., 2022). In contrast to the above findings, our study demonstrated that ADHD group no notable distinctions that of control group in all areas. In consideration of the fact that the changes of GWC are determined by many factors such as white matter and gray matter, whether changes in cortical thickness can affect GWC needs to be further explored in future studies.

In addition, we also found that the GWC of the left transverse temporal was negatively correlated with the degree of inattention in children with ADHD. These results suggested that in male children with ADHD, the decreased GWC of the left transverse temporal indicated that the degree of inattention may increase. The structural and functional abnormalities of the left transverse temporal may be related to the progression of ADHD. However, this study did not find a correlation between GWC and scores for Hyper-Impulsive or ADHD- Index in male children with ADHD. This may be due to the fact that among the 43 ADHD children, 16 were ADHD-C, 1 was ADHD-H, 26 were ADHD-I, the number of ADHD-attention deficit type children was relatively large.

The present study has several limitations. First, only male children were included in this study because it is not clear whether gender differences would affect the brain structure and function of children. We did not conduct in-depth research and discussion on female children with ADHD. Second, this study is a small sample study. Although the ADHD-200 database contains MRI images and patient information were collected by 8 centers, in order to reduce the differences in instruments and parameters among various centers, only the dataset samples of Peking University were used in this study. In addition, in order to exclude the influence of other diseases on this study, we only included children with a single ADHD disorder, so the sample size was screened again. Third, we did not consider the effect of medications on ADHD, because some of the patients included in our study have already received medications. Study has found that compared with healthy control children and ADHD children who have received medication, the white matter volume decreased more obviously in ADHD children who have not received medication (Castellanos et al., 2002). And a recent study found that ADHD children who were treated with methylphenidate for 4 months had increased FA values in the corpus callosum compared to those untreated ADHD children (Bouziane et al., 2019). However, study also found that ADHD children treated with drugs had no changes in white matter microstructure (Bouziane et al., 2018). Fourth, an analysis of subtypes of ADHD was lacking in this study. The three subtypes of ADHD are different in anatomy and function. So far, little is known about the microstructural changes of gray and white matter in specific regions of the three subtypes. Further studies are needed in the future to better understand the differences between ADHD subtypes. Fifth, there are many influencing factors related to GWC, which cannot be analyzed unilaterally from cortical thickness and the degree of myelination of gray and white matter, and further studies are needed to discuss this in the future. Sixth, the questionnaires used in this study were obtained from a public database, and the variety and associated information of these questionnaires were limited. Seventh, there is a lack of information regarding the duration of illness in the patients. Eighth, due to the limited sample size of male children with ADHD, no multiple comparison correction was performed in the correlation study. Applying stringent correction methods may potentially obscure genuine effects.

## 5 Conclusion

To sum up, our study underscores the heightened GWC in bilateral lingual, bilateral insular, left transverse temporal, right parahippocampal and right pericalcarine regions among male children with ADHD, as compared to the healthy controls. We postulate that alterations in cortical myelin, gray matter volume, and white matter microstructure contribute to signal changes in gray and white matter, influencing GWC. In children with ADHD, the GWC of the left transverse temporal might mirror inattention severity. This holistic assessment via GWC aids ADHA diagnosis and treatment evaluation by capturing brain structure and function more comprehensively.

## Data availability statement

The original contributions presented in this study are included in this article/Supplementary material, further inquiries can be directed to the corresponding author.

## Ethics statement

The studies involving humans were approved by the Research Ethics Review Board of Institute of Mental Health, Peking University. The studies were conducted in accordance with the local legislation and institutional requirements. Written informed consent for participation in this study was provided by the participants’ legal guardians/next of kin. Written informed consent was obtained from the individual(s) and minor(s)’ legal guardian/next of kin, for the publication of any potentially identifiable images or data included in this article.

## Author contributions

CW: Writing – original draft, Writing – review & editing. YS: Software, Writing – review & editing. MC: Conceptualization, Data curation, Writing – review & editing. ZZ: Data curation, Investigation, Validation, Writing – review & editing. YL: Conceptualization, Data curation, Writing – review & editing. XiaZ: Methodology, Project administration, Writing – review & editing. ZF: Data curation, Formal analysis, Writing – review & editing. ZY: Formal analysis, Project administration, Writing – review & editing. XinZ: Conceptualization. Writing – review & editing.

## Footnotes

1
http://fcon_1000.projects.nitrc.org/indi/adhd200/
